# *AP4B1*-associated hereditary spastic paraplegia: expansion of phenotypic spectrum related to homozygous p.Thr387fs variant

**DOI:** 10.1007/s13353-020-00552-w

**Published:** 2020-03-12

**Authors:** Krzysztof Szczałuba, Hanna Mierzewska, Robert Śmigiel, Joanna Kosińska, Agnieszka Koppolu, Anna Biernacka, Piotr Stawiński, Agnieszka Pollak, Małgorzata Rydzanicz, Rafał Płoski

**Affiliations:** 1 Department of Medical Genetics, Medical University of Warsaw, ul. Pawinskiego 3c, 02-106 Warsaw, Poland; 2 Department of Child and Adolescent Neurology, Institute of Mother and Child, Warsaw, Poland; 3 Department of Paediatrics, Division of Paediatric Propaedeutics and Rare Disorders, Wroclawa Medical University, Wroclaw, Poland; 4 Postgraduate School of Molecular Medicine, Warsaw, Poland; 5 Department of Genetics, Institute of Physiology and Pathology of Hearing, Warsaw, Poland

**Keywords:** Hereditary spastic paraplegia, Neurodevelopmental disorder, *AP4B1*, Exome sequencing

## Abstract

Biallelic mutations in the *AP4B1* gene, encoding adaptor-related protein complex 4 beta-1 subunit, have been recognized as an important cause of a group of conditions leading to adaptor-related protein complex 4 (AP4)-associated hereditary spastic paraplegia (SPG47). We describe a homozygous, known variant c.1160_1161delCA (p.Thr387fs) that was found in the largest ever group of patients coming from four families. The patients exhibited early hypotonia progressing to spastic paraplegia, microcephaly, epilepsy, and central nervous system (CNS) defects and global developmental delay that are consistent with the nature of SPG47. Our findings expand phenotypic spectrum of SPG47 to include polymorphic seizures, mild/moderate intellectual disability, and intracerebral cysts as well as point to founder mutation in AP4 deficiency disorders in apparently non-consanguineous Polish families without shared ancestry.

**Electronic supplementary material:**

The online version of this article (10.1007/s13353-020-00552-w) contains supplementary material, which is available to authorized users.

## Introduction

The pathogenesis of a number of human disorders has been associated with inefficient bidirectional trafficking of proteins between Golgi apparatus and endosomes (Progida and Bakke 2016). Specifically, the causative link has been established between trafficking apparatus mutations and neurodegenerative conditions, including lysosomal storage disorders, Alzheimer’s and Parkinson’s disease, and postnatal microcephaly phenotype (Schreij et al. 2016; Passemard et al. 2017).

The number of proteins involved in a proper function of the so-called trans-Golgi network (TGN) is abundant. One of these, adaptor-related protein complex 4 beta-1 subunit (AP4B1), is part of the tetrameric AP-4 complex that is expressed ubiquitously in human tissues, including central nervous system (Dell'Angelica et al. 1999; Hirst et al. 1999). *AP4B1* pathogenic variants lead to a rare but well-recognized hereditary spastic paraplegia type 47 (SPG47) phenotype (Abou Jamra et al. 2011; Blumkin et al. 2011; Bauer et al. 2012; Ebrahimi-Fakhari et al. 2018a).

Herein, we present five patients from four families with recurrent homozygous c.1160_1161delCA (p.Thr387fs) *AP4B1* variant. All the patients present with hypotonia progressing to spastic paraplegia, microcephaly (4 out of 5), epilepsy (4 out of 5), central nervous system (CNS) defects (4 out of 5), and global developmental delay. Our observations confirm and broaden the clinical spectrum of AP-4-associated hereditary spastic paraplegia and point to a founder mutation in apparently non-consanguineous families without shared ancestry.

## Clinical report

### Family 1 (patients 1 and 2)

The patients were two sisters of unrelated parents. First girl, the older one, was born after uneventful pregnancy and delivery with weight 3600 g, occipitofrontal circumference 34 cm, and Apgar score of 10 points. Her psychomotor development was delayed. Since 9 months of age, epileptic polymorphic seizures have started and have been successfully treated with valproic acid and lamotrigine. Due to hypotonia, she started to walk independently at age 3 years and her gait was clumsy, on tiptoes. She started to talk at the same age, and her speech was blurred and dysarthric. At age 5 years, neurological examination revealed spastic paraparesis with elevated tendon reflexes and bilateral Babinski signs. Despite the negative medical history of perinatal hypoxia, cerebral palsy was diagnosed.

The younger sister was born after uneventful pregnancy and delivery with weight 3300 g, occipitofrontal circumference (OFC) 34 cm, and Apgar 9 points due to hypotonia. Her psychomotor development was delayed, and at age 9, polymorphic seizures started, mainly during febrile infection. Valproic acid was administered with good effect. She started to walk at age 2 years with abnormal movement pattern—she would fall down frequently. At 3 years, spastic paraparesis was noted.

**Fig. 1:**
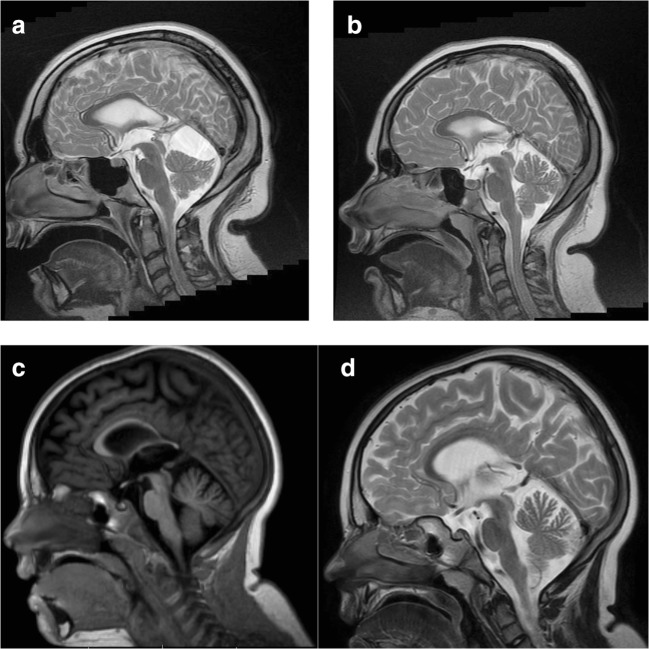
**a** MRI image of patient 1 (family 1), sagittal section. In T2-weigted image, thinning of the posterior part of corpus callosum is visible as well as an infratentorial cyst modeling the cerebellum. See also brachycephaly due to small brain. **b** MRI image of patient 2 (family 1), sagittal section. In T2-weighted image, thinning of a posterior part of corpus callosum is visible. See also brachycephaly due to small brain. **c**, **d** MRI of patient 3 (family 2), sagittal section. In T1-weigted image, the similar thinning of the posterior part of corpus callosum is visible. In this patient, a relative white matter loss can also be recognized in a T2 sagittal section

The girls were both admitted to our Neurologic Department at the ages 11 and 6 years, respectively, due to a suspicion of familial spastic paraparesis. Older girl was wheelchair-bound, while the younger walked alone but her gait pattern was abnormal. Both were in good emotional and social contact. On physical examination, their internal organs were normal. Neurological examination revealed spastic paraparesis and bilateral Babinski sign. Ophthalmologic examination showed normal appearance of the optic nerves disk without features of intracranial hypertension. Routine laboratory tests showed no abnormalities. Electroencephalogram (EEG) showed abnormal generalized discharges over both hemispheres. Repeated magnetic resonance imaging (MRI) in both girls revealed normal myelination of cerebral white matter, but their corpus callosum was markedly thinned especially in its posterior part. Additionally both girls had arachnoid cysts. The older sister had a cyst in the area of the right side of Sylvian fissure of the size 51 x 37 x 85 mm with mass effect: the right lateral ventricle was compressed and the midline structures were displaced to the left side by about 4.7 mm (Fig. 1a). The younger sister had a smaller cyst 18 x 20 x 21 mm located in the midline under the tent of the cerebellum without mass effect (Fig. 1b). Neuropsychological examinations performed with Leiter International Performance Scale showed moderate mental disability in the older sister and a mild one in the younger.

Now, at ages 22 and 17, their growth parameters are 148 cm (below 3c) and 150 cm (below 3c) and OFCs are 52 cm (below 3c) and 52.5 cm (about 3c), respectively. Both are overweight and wheelchair-bound. Neurosurgery performed in the older sister at age 13 due to the cyst mass effect was not affected by her neurological and general condition.

### Family 2 (patient 3)

The proband boy was born to non-consanguineous parents. He had two younger healthy sisters. No family history of note or exposure to teratogens was reported. The proband was born at 39/40-week gestation. Apgar scores were 8 after a minute and 10 after 5 min. Birth weight was 3100 g (10–50c), length 54 cm (97c), and OFC 35 cm (50–90c). No congenital anomalies or dysmorphic features were noted at birth. Perinatal screening was normal.

At the neonatal age, the parents noticed generalized hypotonia and open mouth. The sucking reflex was sufficient so that he could be breastfed. Despite intensive rehabilitation, gross motor delay was noted: he sat unsupported at age 14 months and walked unassisted at 30 months. The gait was unstable and eventually, contractures of major joints developed. By age 3, the proband was diagnosed with focal epilepsy that was reactive to standard medication. He remained asymptomatic for the last year. Diagnostic brain imaging was performed twice: at age 3 revealing normal pattern of myelination and apparently no brain anomalies and then at 5 years showing relatively mild white matter loss as well as corpus callosum hypoplasia (Fig. 1c). EEG of the brain showed the presence of abnormal discharges over fronto-temporo-occipital cortex. Metabolic screening was normal, as was the hearing assessment.

Currently, an 8.5-year-old proband presents with the predominant picture of non-progressive spastic paraplegia. He can move with a walker, having experienced a number of lower limb surgeries. As the proband is able to speak merely single words, severe speech delay has been recognized. His current weight is 31 kg (75–85c), height 125 cm (15–25c), and occipitofrontal circumference 52 cm (75–90c).

### Family 3 (patient 4)

The proband girl was born from the second pregnancy to non-consanguineous parents. No family history of note or exposure to teratogens was reported. The proband was born at 41-week gestation. Apgar scores were 10 after 1 and 5 min. Birth weight was 3650 g (50c), length 56 cm (97c), and OFC 35 cm (50c). No congenital anomalies or dysmorphic features were noted at birth. Perinatal screening was normal.

At age 2 months, the parents noticed weak response to external stimuli. Later, gross motor delay was noted: she sat unsupported at age 8 months and walked unassisted at 24 months. Typical pattern of tonus abnormalities was noted: at first, she was hypotonic, with increased muscle tone following, that resulted in abnormal gait: stiff legs with femurs internally rotated and knees extended. Botulinum toxin was attempted a number of times to relieve spasticity. She had severe speech delay. She experienced a single episode of febrile seizures at age 8 months. She remained asymptomatic for the last year. Diagnostic brain imaging revealed corpus callosum hypoplasia (Fig. 1c). EEG of the brain showed the presence of abnormal discharges over the left temporo-occipital cortex. There was no hearing impairment.

Currently, a 10-year-old proband presents with non-progressive spastic paraplegia. She can move with assistance. She is severely delayed and can only understand simple commands being unable to control physiological needs. Her current weight is 35 kg (50c), height 122 cm (< 3c), and occipitofrontal circumference 50 cm (< 3c).

### Family 4 (patient 5)

The proband boy was born from the first twin dizygotic pregnancy to non-consanguineous parents. No family history of note or exposure to teratogens was reported. The proband was born at 38-week gestation after uneventful prenatal period. Apgar scores were 9 and 10 after 1 and 5 min, respectively. Birth weight was 3600 g (50–90c), length 55 cm (97c), and OFC 34 cm (50c). No congenital anomalies or dysmorphic features were noted at birth. Perinatal ultrasound screening was normal. Hearing test was normal.

Psychomotor development was delayed. During the first 12 months of age, the proband was hypotonic, he sat at age 9 months and walked with assistance at 18 months. Increased muscle tone in lower extremities was observed from 18 months of age that resulted in abnormal stiff gait. Botulinum toxin injections were attempted three times as a treatment of spasticity. He had severe speech delay; up to date, he speaks merely several words. At age 18 months, a single episode of febrile seizures was observed, but no epilepsy was diagnosed. Magnetic resonance of the brain did not reveal any abnormalities, but EEG showed the presence of abnormal discharges over fronto-temporal cortex.

Currently, a 12-year-old boy presents with non-progressive spastic paraplegia. He can move only with assistance, and he is unable to control his physiological needs. Severe intellectual disability is diagnosed. His current weight is 50 kg (75c), height 150 cm (25–50c), and occipitofrontal circumference 51 cm (< 3c). Apart from microcephaly, no dysmorphic facial features are observed.

### Genomic testing

DNA from all patients and their family members was extracted from the peripheral blood with a standard protocol. The DNA from patient1 (family 1) was analyzed by clinical exome sequencing (TruSight One Sequencing Panel (Illumina Inc., San Diego, CA, USA), TSO), which provides comprehensive coverage of over 4800 disease-associated genes. For the remaining patients, the whole exome sequencing (WES) was performed. Library preparation for WES was prepared with the following commercially available kits: (i) patient 3 from family 2—SureSelectXT Human All Exon v5 (Agilent, Santa Clara, CA, USA), (ii) patient 4 from family 3—SeqCap EZ MedExome (Roche, Basel, Switzerland), and (iii) patient 5 from family 4—SureSelectXT Human All Exon v7 (Agilent). Subsequently a paired-end sequencing was performed on HiSeq 1500 to the mean depth of at least 60×. A total of 90% or more of target bases were covered at a minimum of 20×, whereas 96% had coverage of min.10×. The obtained raw data was analyzed as previously described (Ploski et al. 2014) with hg19 genomic build used for alignments. All variants of interest were inspected with Integrative Genomics Viewer (IGV).

**Fig. 2:**
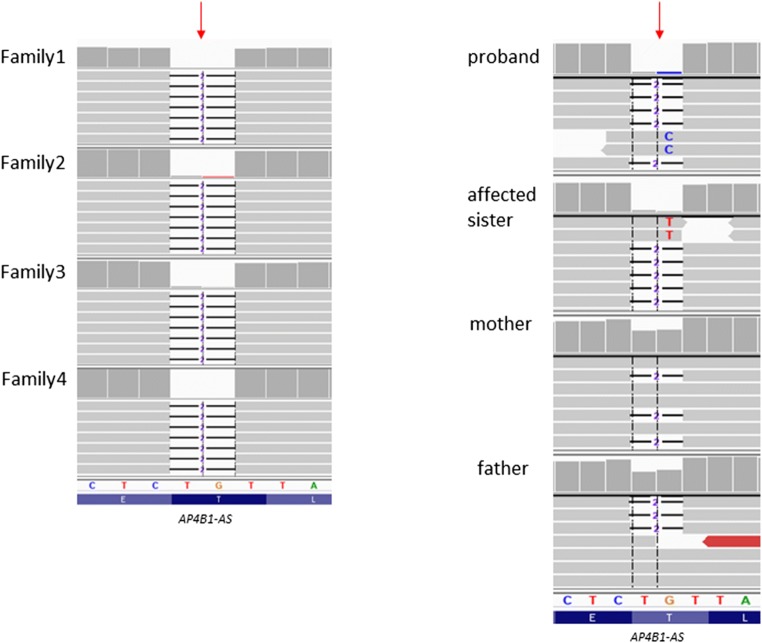
Results of WES in patients from families 1–4 (left panel), example image of the results of amplicon deep sequencing in patients 1 and 2 and their relatives (right panel) from family 1

Considering the patients’ phenotype for further analysis, we prioritized a homozygous variant located within *AP4B1* gene: NM_006594.4:c.1160_1161delCA is predicted to result in p.Thr387Argfs, which has been previously reported as causative of hereditary spastic paraplegia type 47 (Abdollahpour et al. 2015; Ebrahimi-Fakhari et al. 2018a, b). Presence of the c.1160_1161delCA variant was verified by amplicon deep sequencing (NGS analysis of an appropriate PCR amplicon, coverage > 500×) in all the patients and their parents, thus confirming an in trans configuration (Fig. 2).

The population frequency for the c.1160_1161delCA variant was 0.00014 in gnomAD database (v.2.0.2, https://gnomad.broadinstitute.org/), 0.00018 in ClinVar (https://www.ncbi.nlm.nih.gov/clinvar/), and 0.0027 in an in-house database of > 3000 WES of Polish individuals.

All probands’ parents declared lack of consanguinity. Independently, we performed re-analysis of TSO/WES data for runs of homozygosity (ROH) as previously described (Śmigiel et al. 2018). Patient 1 was in the 38th percentile for total ROH length (533 out of 1387), patient 3 was in the 27th percentile for total ROH length (215 out of 796), patient 4 was in the 33rd percentile for total ROH length (157 out of 468), and patient 5 was in the 85th percentile for total ROH length (416 out of 489). Thus, there was no evidence for consanguinity between parents of our patients. The results of ROH analysis are shown in Supplementary Figure 1.

## Discussion

We describe five individuals from four families in whom a homozygous c.1160_1161delCA (p.Thr387fs) variant in the *AP4B1* gene was found, which led to the diagnosis of hereditary spastic paraplegia type 47 (SPG47) (Abdollahpour et al. 2015; Ebrahimi-Fakhari et al. 2018a, b).

The common phenotypic features seen in all the five affected children were global developmental delay, including severe speech delay; hypotonia progressing to spastic paraplegia; and epilepsy/seizures. Microcephaly or CNS anomalies were present in four patients. The growth of all patients from families 1 and 3 was stunted. Abdollahpour et al. (2015) presented a similar phenotype of SPG47 caused by the same frameshift variant (Abdollahpour et al. 2015). Most features, including a rather non-specific callosal hypoplasia resulting from a reduced volume of white matter and a more typical pattern of progression from early hypotonia to paraplegia, were almost identical to those observed in our patients. Thinning of corpus callosum in its posterior part and a gradual white matter loss may explain mainly lower limb involvement in SPG47 and a typical progression of the disease. Detailed neurological examination when hypotonia is present, and a later follow-up to search for paraplegia/spasticity coupled with serial MRI, is perhaps the key to the diagnosis of all AP-4 disorders. Thereby, they could easily be differentiated from cerebral palsy that has a very slow non-progressive course. It is worth noting that the proband from family 1 had been misdiagnosed as cerebral palsy until the second affected child was born within the same family. Unfortunately, to this day, in the cases of early-onset neurodegenerative diseases, a number of genetic conditions are being mislabeled as cerebral palsy.

Typically for SPG47, EEG was abnormal in all presented cases. However, in our patients, polymorphic seizures (tonic, tonic-clonic, and focal generalizing) were diagnosed, which broadens the spectrum of epilepsy phenotypes in SPG47. A never-reported finding is also a relatively mild to moderate intellectual impairment recognized in family 1 versus severe one noted in previous SPG47 patients. Both patients from family 1 presented with intracerebral cysts. Cysts are very rarely observed in spastic paraplegia individuals except SPG4 (posterior fossa findings) and SPG11 (Orlacchio et al. 2004; Abdel Aleem et al. 2011). Their pathomechanism is currently unknown but may be linked to white matter loss. We did not find any growth pattern abnormalities or dysmorphism in our patients. A more detailed clinical comparison between our cases and those reported previously is presented in Table 1.

**Table 1:** Comparison of clinical features of the exact same mutation in *AP4B1* between our cases and cases reported by Abdollahpour et al. (2015) and Ebrahimi-Fakhari et al. (2018a)

Feature	Family 1 sibling 1	Family 1 sibling 2	Family 2	Family 3	Family 4	Abdollahpour et al. (2015) (sibling 1/sibling 2)	Ebrahimi-Fakhari (2018) (patient 3—compound heterozygote)
Age at presentation (years)	22	17	8.5	10	12	14/12	2.5
Head circumference	Microcephaly	Microcephaly	Normal	Microcephaly	Microcephaly	Microcephaly/microcephaly	Microcephaly
Short stature	+	+	−	+	−	+/+	+
Dysmorphism	No	No	No	No	No	Yes/yes	?
ID/dev delay	Moderate	Mild	Severe	Severe	Severe	Severe/severe	Moderate
Seizures/epilepsy	+	+	+	+	Febrile	Febrile/febrile	Febrile
Speech	Dysarthric	Dysarthric	Severely delayed	Non-verbal	Severely delayed	Non-verbal/non-verbal	Non-verbal
Early hypotonia	+	+	+	+	+	?/?	+
Progression to hypertonia	+	+	+	+	+	+/+	?
Hyperreflexia/spasticity	+	+	+	+	+	+/+	−
Head MRI abnormalities	+	+	+	+	−	−/+	+
Other features	none	None	None	none	None	Clubfoot/clubfoot	None
Independent walking	3 years	2 years	2.5 years	2 years	18 months	20 months/18 months	35 months
Ambulation	Wheelchair	Wheelchair	Walker	With assistance	With assistance	Wheelchair/wheelchair	With assistance

The AP-4 complex sorts transmembrane cargo proteins into transport vesicles for trans-Golgi network export. A defect in only one subunit of AP-4 (e.g., AP4B1) leads to an abnormal function of the whole complex. The majority of pathogenic variants in *AP4B1* are frameshift or nonsense as they lead to nonsense-mediated RNA degradation (Hirst et al. 2013). Variant detected in our families is also a frameshift and thus far, together with the c.664delC (p.Leu222Cysfs*31) variant reported by Ebrahimi-Fakhari et al., it is the most common *AP4B1* mutation (Ebrahimi-Fakhari et al. 2018a). The high population prevalence may be due to either a founder effect or the presence of a mutational hotspot. For the c.1160_1161delCA, we suggest presence of a founder effect. This is supported by the fact that the variant is not located in a region with motifs predisposing to occurrence of the mutations (analysis performed with non-B DNA Motif Search Toolhttps://nonb-abcc.ncifcrf.gov/apps/nBMST). Furthermore, c.1160_1161delCA is found on a shared haplotype (Supplementary Table 1) despite the lack of evidence for consanguinity of patients’ parents. Finally, the variant has a high carrier frequency in Poland. In particular, c.1160_1161delCA allele frequency is more than 10 times higher in the database of WES variants representative of the Polish population than in other publicly available databases, which suggests founder effect in Polish/Eastern European populations.

In conclusion, our report broadens the phenotypic spectrum of AP4B1-associated neurologic disease to include polymorphic seizures, mild/moderate intellectual disability, and intracerebral cysts and points to a founder effect in apparently non-related Europeans with a uniform phenotype resulting from the same homozygous variant. We suggest that variants in *AP4B1* gene be included in next-generation sequencing panels designed for epilepsy, intellectual disability/developmental delay, spastic paraplegia, and central nervous system malformations.

## Electronic supplementary material

ESM 1 (DOCX 66 kb)
