# Supplementary material for: AP4B1-associated hereditary spastic paraplegia: expansion of phenotypic spectrum related to homozygous p.Thr387fs variant
Source: J Appl Genet. 2020 Mar 12;61(2):213–8. doi: 10.1007/s13353-020-00552-w (PMC7148264; doi:10.1007/s13353-020-00552-w)
Supplement: Supplementary file 1 — (DOCX 66 kb) [file 13353_2020_552_MOESM1_ESM.docx]

**Supplementary Table 1**. Genotypes of selected SNPs in close proximity of the c.1160_1161delCA (p.Thr387fs) variant. Note in particular the uniform homozygosity at a neighboring marker at chr.1:113 704 824

|  | chr. position (hg38) | | | | | | | | | |
| --- | --- | --- | --- | --- | --- | --- | --- | --- | --- | --- |
| Patient | 1:111 483 827 (MAF*=0.104) | 1:111 691 462 (MAF=0.123) | 1:112 712 461 (MAF=0.008) | 1:112 724 147 (MAF=0.047) | 1:113 704 824 (MAF=0.084) | **1:113 898 754 (MAF=0.0001)** | 1:114 510 877 (MAF=0.065) | 1:114 601 414 (MAF=0.041) | 1:114 673 772 (MAF=0.006) | 1:115 664 330 (MAF=0.118) |
| **1** | A/- | nd | nd | nd | nd | **delTG/delTG** | nd | nd | -/- | nd |
| **3** | A/- | -/- | A/- | C/- | A/A | **delTG/delTG** | -/- | A/- | -/- | -/- |
| **4** | A/- | T/- | A/A | C/- | A/A | **delTG/delTG** | G/- | A/- | T/- | G/- |
| **5** | -/- | T/- | A/A | C/C | A/A | **delTG/delTG** | -/- | A/A | -/- | G/G |

* MAF (Minor allele frequency according to gnomAD database https://gnomad.broadinstitute.org/); nd = no data (due to the restricted target of TSO);
 “ –“ denotes ancestral allele, c.1160_1161delCA (p.Thr387fs) variant in *AP4B1* is bolded

**
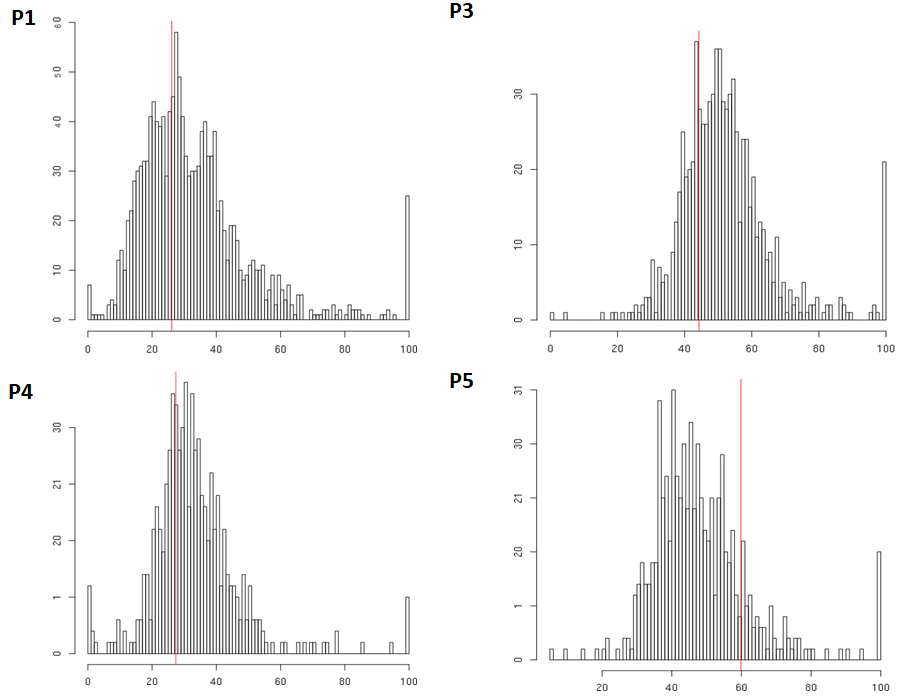
Supplementary Figure 1**. Comparison of total ROH length. Horizontal axis shows total ROH length in Mb, vertical axis indicates number of individuals in database analyzed with the same methodology. **P1** – patient 1 and 1387 unrelated Polish individuals analyzed by TruSight One Sequencing Panel target, **P3** - patient 3 and 796 unrelated Polish individuals analyzed by SureSelectXT Human All Exon v5 target, **P4** - patient 4 and 468 unrelated Polish individuals analyzed by SeqCap EZ MedExome target, **P5** - patient 5 and 489 unrelated Polish individuals analyzed by SureSelectXT Human All Exon v7 target. Red bars indicate the total ROH length of particular patients.
